# Toxic epidermal necrolysis following heart transplantation may caused by cefoperazone sodium and sulbactam sodium

**DOI:** 10.1186/s13019-024-03025-x

**Published:** 2024-09-23

**Authors:** Zeng Xiaodong, Wu Min, Lei Liming, Huang Jinsong, Qi Xiao, Liang Yuemei, Wu Yijin

**Affiliations:** 1 Department of Cardiac Surgery Intensive Care Unit, Guangdong Cardiovascular Institute, Guangdong Provincial People’s Hospital (Guangdong Academy of Medical Sciences), Southern Medical University, Guangzhou, 510080 China; 2 Department of Cardiac Surgery, Guangdong Cardiovascular Institute, Guangdong Provincial People’s Hospital (Guangdong Academy of Medical Sciences), Southern Medical University, Guangzhou, Guangdong 510080 China; 3 Guangzhou Health Science College, Guangzhou, Guangdong 510000 China

**Keywords:** Heart transplantation, Immunosuppression, Drug-induced dermatitis, Toxic epidermal necrolysis, Cefoperazone sodium and sulbactam sodium

## Abstract

**Background:**

The outcome of heart transplantation is significantly affected by perioperative infections. Individualised immunosuppression strategies are essential to reduce the risk of such infections.

**Case presentation:**

We report the successful management of a 56-year-old male patient diagnosed with dilated cardiomyopathy who underwent heart transplantation. During the perioperative period, the patient was prescribed cefoperazone sodium and sulbactam sodium, which induced a severe skin reaction: toxic epidermal necrolysis (TEN). The patient was treated with prednisone, immunoglobulins, etanercept, and other active immunomodulatory measures, together with an individualised anti-rejection regimen and physical therapy. The systemic rash resolved within a month, and the patient was successfully discharged after surgery.

**Conclusion:**

Effective management of heart transplantation necessitates balancing immunosuppression and infection prevention. Individualised immunosuppressive strategies are critical for optimal clinical outcomes.

## Introduction

Heart transplantation is the most effective treatment for end-stage heart failure. However, perioperative immunosuppressive treatment, infections and drug side effects remain significant factors limiting surgical success. Individualised immunosuppression strategies and effective infection prevention are current research hotspots in the field of heart transplantation [1, 2]. Toxic epidermal necrolysis (TEN), a rare yet serious skin exfoliation disease, has garnered clinical attention in recent years [3].

TEN is often triggered by drug allergy, especially antibiotics used in the perioperative period [4]. The disease is characterised by an acute onset, extensive skin exfoliation, and systemic involvement, which poses a major challenge to patients’ recovery and survival. Although the exact pathogenesis of TEN is not fully understood, early diagnosis and aggressive treatment strategies are key to reducing mortality [3, 5].

While immunosuppressive treatment regimens reduce post-transplant rejection, they also increase the patient’s risk of infection and other complications. Therefore, how to effectively balance immunosuppression and immune monitoring, and how to adjust treatment plans for complications, has become a challenge for clinicians. This article reports an extremely rare case of drug-induced TEN in a 56-year-old male patient who underwent heart transplantation. The aim of this case report is to explore the mechanisms, clinical manifestations, diagnosis and treatment of TEN after heart transplantation and to provide relevant suggestions for the management of immune rejection after transplantation.

## Case presentation

A 56-year-old male patient was admitted to the Guangdong provincial People’s Hospital (Guangzhou, China) in September 2023 for severe heart failure. One year before admission, the patient experienced symptoms of palpitations and shortness of breath, which were relieved by rest without receiving standardised treatment. One month prior to admission to our hospital, his symptoms worsened and paroxysmal dyspnea occurred at night. His past medical history includes diabetes mellitus and hypertension. The patient was on optimal heart failure medication, including ARBs, beta-blockers, diuretics, aldosterone blockers, etc.

**Fig. 1:**
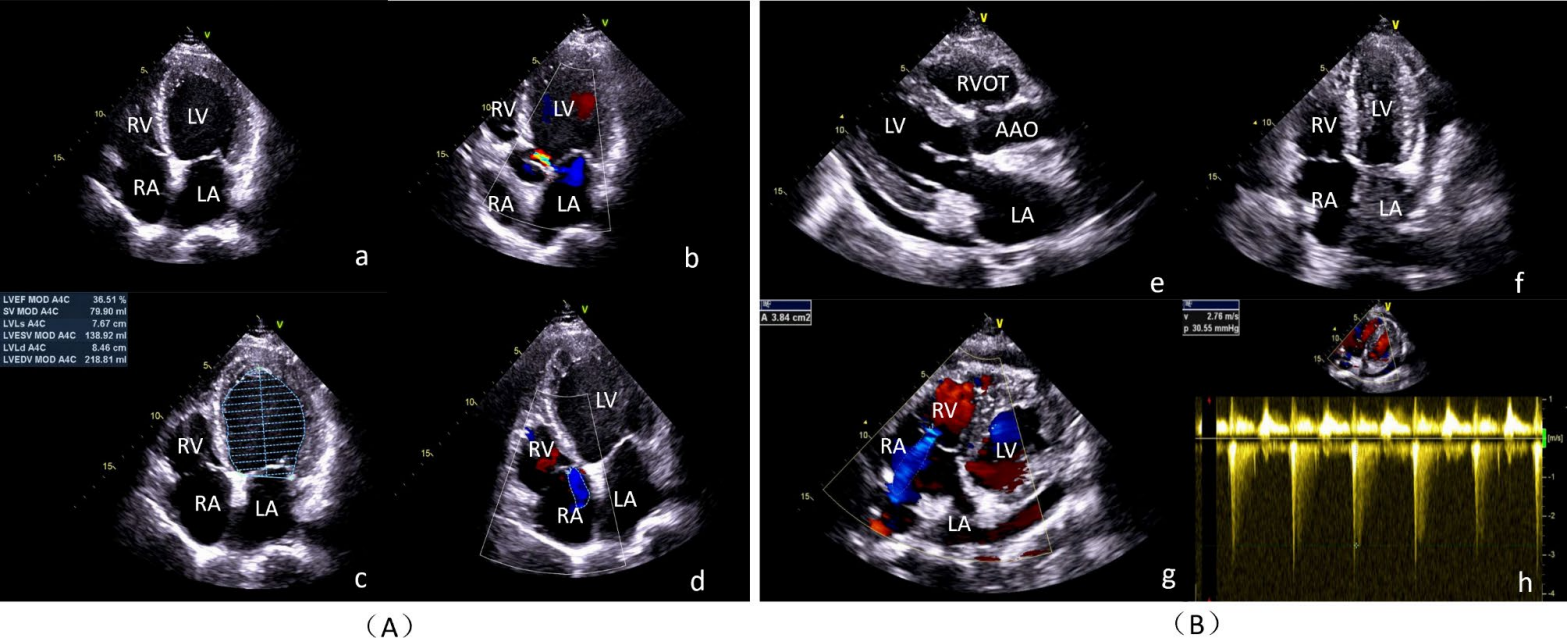
Ultrasound Doppler before (**A**) and after(**B**) heart transplantation. LV: left ventricle; LA: left atrium; RV: right ventricle; RA: right atrium; RVOT: right ventricular outflow tract; AAO: ascending aorta; LVEF: left ventricular ejection fraction

On admission, his body temperature was 37 °C, with heart rate 44 beats per minute, blood pressure of 100/65mmHg, respiratory rate 20 breaths per minute. His height is 170 cm and weight is 65.5 kg. Echocardiography revealed dilated cardiomyopathy, with a left ventricular end diastolic diameter of 65 mm and an estimated left ventricular ejection fraction (LVEF) of 36.5% (Fig. 1). A whole aorta CT scan showed no significant coronary artery stenosis. Cardiopulmonary exercise test results showed that a peak exercise oxygen consumption (VO_2_) was 13.3 ml/min/kg, accounting for 42%pred, and a ventilation versus carbon dioxide production (VE/VCO_2_) slope of 49.5. Right heart catheterisation showed a pulmonary artery pressure of 26/9mmHg, total pulmonary resistance of 4.9 wood units and small pulmonary resistance of 2.1 wood units. On admission, he had erythema on the face, trunk and extremities, but his medication history prior to the rash was unfortunately unknown. Dermatology consultation considered drug-induced dermatitis, erythema multiforme, and the patient was treated with methylprednisolone 40 mg/day for 4 days on specialist advice. The rash was stable. After a multidisciplinary meeting, he was approved and listed for heart transplantation.

## Surgical procedure

### Procurement of donor heart

The donor heart was obtained from a brain-dead patient. Following a silent tribute to the donor, a thoracotomy was performed. A purse-string perfusion needle was sutured onto the ascending aorta, and Modified St. Thomas Solution was perfused into the aortic root to carefully arrest the heart. Subsequently, the superior and inferior vena cava, posterior wall of the left atrium, aorta, and pulmonary artery were isolated respectively to obtain a donor heart. HTK perfusion was performed after isolation.

### Heart transplant process

The heart transplant was performed using the bicaval technique. Following anesthesia, a mid-sternal thoracotomy was conducted and cardiopulmonary bypass was established. Then, the aorta, superior and inferior vena cava were crossclamped, and the patient’s heart was removed. Simultaneously, the donor heart was sutured immediately. Finally, bleeding was addressed, extracorporeal circulation was routinely removed, and the chest was closed. The cold ischemia time was 139 min, cardiopulmonary bypass time was 255 min and aortic cross-clamp time was 100 min.

### Postoperative changes and management

During the early post-transplant period, the patient’s hemodynamics were stable, and ventilator support was required to maintain respiratory function. On the day after surgery, the patient was administered epinephrine (0.048 µg/kg/min) and milrinone (0.3 µg/kg/min) as vasoactive drugs. Cefoperazone sodium and sulbactam sodium, linezolid, and voriconazole were used for infection prophylaxis. Immunosuppressive therapy commenced shortly after heart transplantation, including the sequential administration of prednisolone, mycophenolate mofetil, and tacrolimus.

**Fig. 2:**
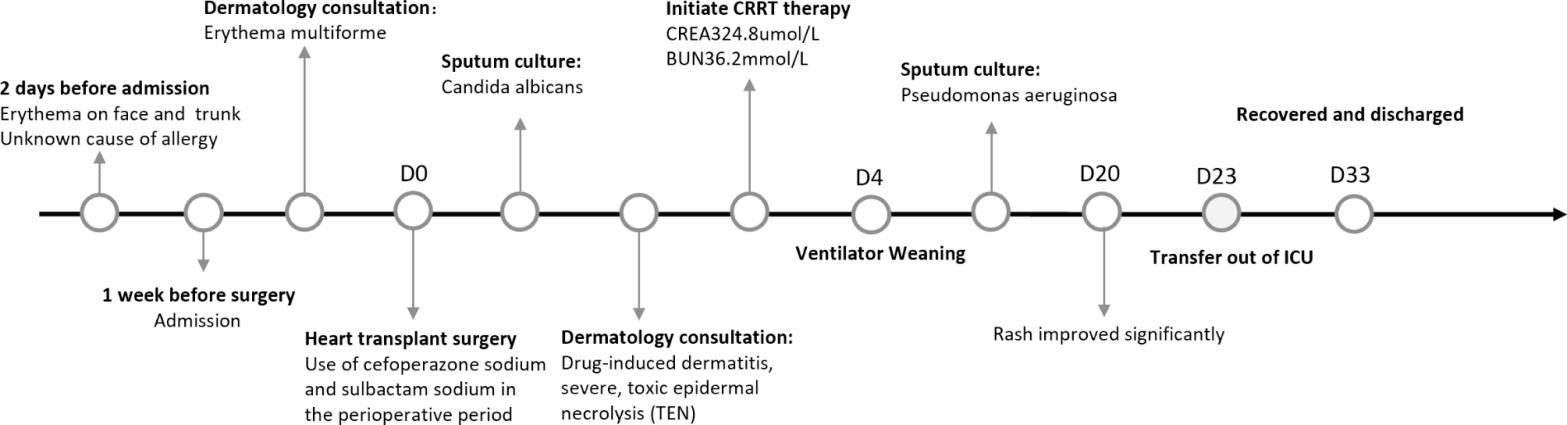
Timeline

**Fig. 3:**
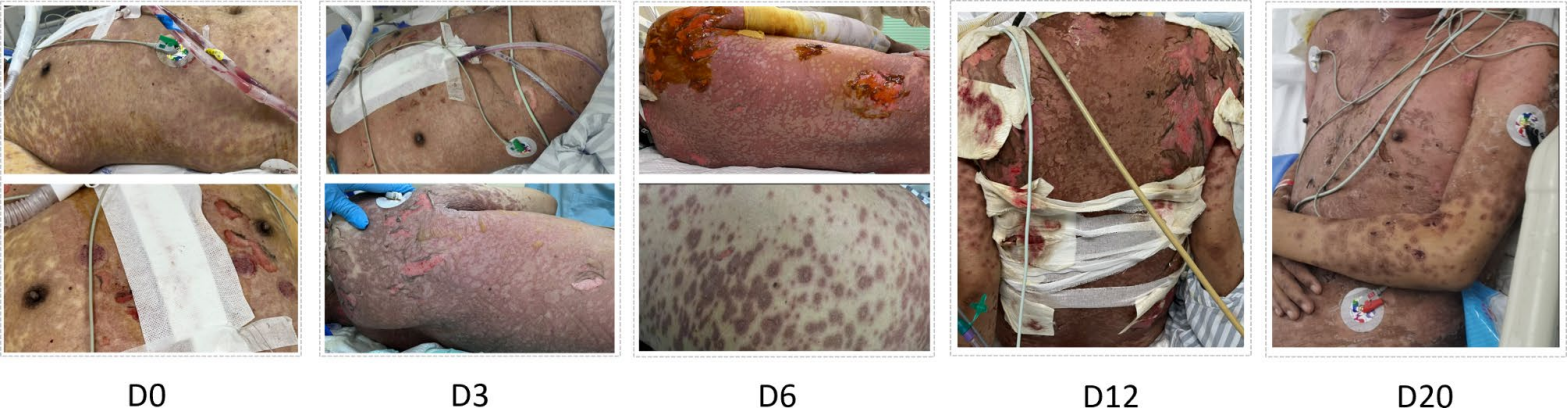
Skin symptoms in patients with toxic epidermal necrolysis. (D0) Rash all over the body. (D3) Exfoliation of skin on the back. (D6) and (D12) Treatment and recovery of patient with toxic epidermal necrolysis. (D20) Skin rash all over the body improved significantly

**Fig. 4:**
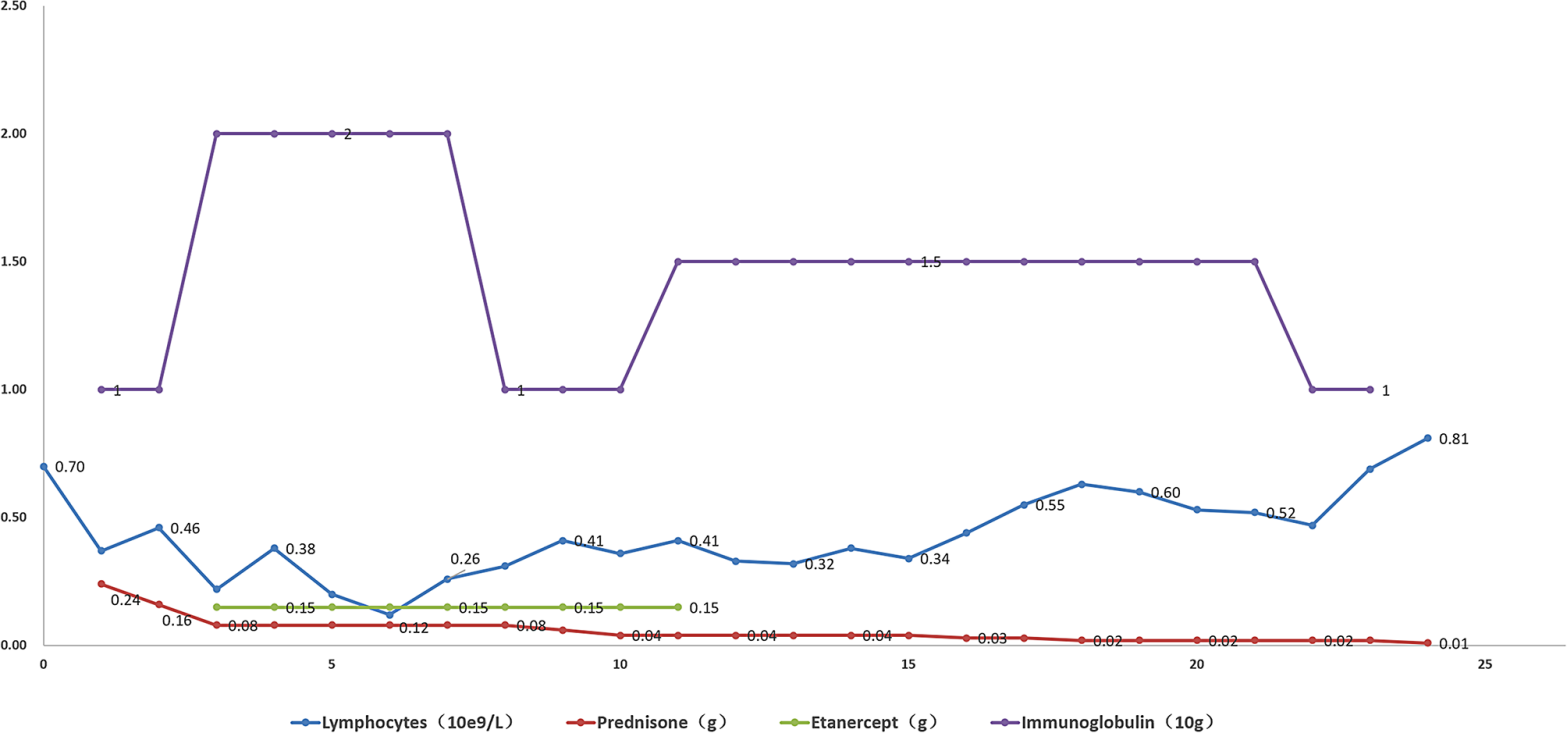
Main therapeutic drugs for toxic epidermal necrolysis at various times after heart transplantation: In the early postoperative period, prednisone, immunoglobulin, and etanercept were used, and in the later period, immunoglobulin was mainly used in combination with low-dose prednisone

The patient’s body rash worsened rapidly on the first day after cardiac surgery (Fig. 2). By the second postoperative day, blisters and bullae appeared on the skin, large areas of epidermis were exfoliated, Nissl’s sign was positive, and the oral cavity, conjunctiva, and mucosa were involved (Fig. 3). Following a dermatology consultation, the patient was diagnosed with severe drug-induced dermatitis, specifically toxic epidermal necrolysis (TEN), and the cause was considered to be related to the use of cefoperazone-sulbactam. A differential diagnosis should be made: Stevens-Johnson syndrome (SJS): Given the overlap in clinical presentation with TEN, it can be distinguished based on the degree of skin sloughing. Pemphigus vulgaris: Clinical presentation and rapid progression of symptoms exclude this autoimmune blistering disease. The patient’s current Toxic Epidermal Necrolysis-specific severity of illness score (SCORTEN) is 5, indicating high mortality risk. Cefoperazone sodium and sulbactam sodium were discontinued, and the patient was switched to meropenem. Subsequently, the patient received treatment with steroids, immunoglobulin, and etanercept (Fig. 4). Vaseline gauze was used to cover the eroded skin surface. Hemodialysis was initiated on the third postoperative day due to elevated serum creatinine levels of 324 umol/L. Sputum culture results indicated moderate Candida albicans and Pseudomonas aeruginosa. Voriconazole was switched to caspofungin, and ceftazidime avibactam sodium was added to the treatment. On the fourth postoperative day, the tracheal intubation was removed smoothly.

By the six days post-surgery, the relaxation and erosion of blisters and bullae had improved, and the erythema became darker. Eleven days after surgery, distinctive dark red skin lesions emerged on the face, upper limbs, and trunk, with blisters and bullae epithelial decomposition and erosion. The eroded surface was dry without exudation, Nissl’s sign was negative, and there was no new rash. The condition improved significantly twenty days after surgery, and the skin lesions were basically healed. He was transferred out of the ICU on the 23rd day post-surgery. By the 25th after surgery, the skin was dry and peeling all over the body. He was successfully discharged 33 days after surgery.

## Discussion

Heart transplantation is the “Gold Standard Therapy” for improving the quality of life and long-term longevity of patients with end-stage heart failure [6]. Postoperative immunosuppressive therapy, aimed at managing potential infections and minimizing drug-related side effects, significantly impacts prognosis. Key principles of immunosuppressive management include combining agents for efficacy, minimizing toxicity, individualizing treatment, and ensuring vigilant monitoring. Current immunosuppressive treatment includes perioperative induction, long-term maintenance, and anti-acute rejection treatment. While specific immunosuppressive agents, dosages, and combinations vary across cardiac centers, overall approaches remain broadly similar. Tailoring immunosuppressive therapy to factors such as recipient age, immune status, and organ function presents a substantial challenge in post-transplant care [1, 2].

This patient experienced a severe epidermolytic necrotic drug eruption attributed to the perioperative use of cefoperazone sodium and sulbactam sodium, an exceptionally rare and serious adverse drug event. Later, the patient’s family struggled to find the medication records of another hospital and found that cefoperazone sodium and sulbactam sodium had been used in another hospital approximately half a month before admission. The patient did not experience any discomfort during that time. The patient had developed a rash two days before admission. Upon admission, chest CT revealed mild inflammation of the right lung, prompting empirical treatment with cefoperazone sodium and sulbactam sodium for three days.

The rash worsened after heart transplantation. After multidisciplinary discussion involving dermatology, pharmacy, and rheumatology, a delayed allergic reaction to cefoperazone sodium and sulbactam sodium was considered. This type of skin reaction is mediated by sensitized T lymphocytes, with rash being the most common clinical manifestation. In severe cases, the reaction can escalate to severe rash and even shock [7–9]. Previous literature reports that factors leading to severe adverse skin reactions in hospitalised patients include a history of allergy, smoking history, and the use of cefoperazone sodium and sulbactam sodium, and levofloxacin, among which antibiotics are the most common causes [10, 11]. The patient tested negative for HLA-I antibodies and suspiciously positive for HLA-II antibodies. He was administered prednisolone 40 mg per day before surgery, stabilizing the rash. Four days later, the patient received the donor heart allocated for heart transplant by the China Organ Transplant Response System (COTRS) system.

**Fig. 5:**
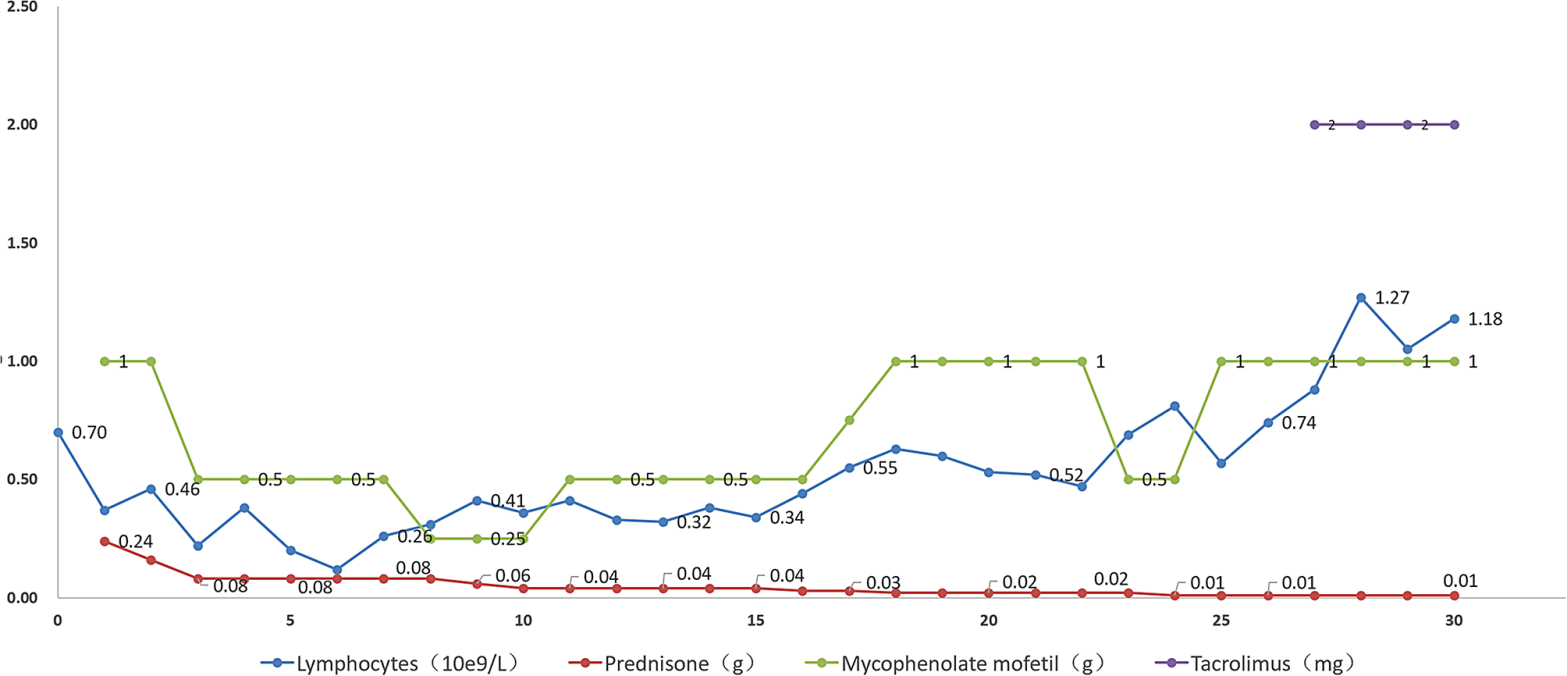
Immunosuppression strategies after heart transplantation: In the early postoperative period, prednisone and mycophenolate mofetil were used. According to the immune status, the dosage of prednisone was slowly reduced, the dosage of mycophenolate mofetil was slowly increased, and tacrolimus was added in the later stage

We implement a comprehensive strategy for the treatment of severe drug-induced dermatitis:① Immediate Discontinuation: Cease cefoperazone sodium and sulbactam sodium and avoid other drugs that may cause allergies. ② We have strengthened our anti-infection strategies to prevent infections caused by skin colonization bacteria such as Staphylococcus epidermidis and Candida albicans. ③ The use of intravenous immunoglobulin to increase IgG levels in the body, neutralise toxins, regulate immune function, synergistically kill bacteria, and prevent infection [12–14];④Etanercept blocks the binding of TNF-α to cell surface TNF receptors, thereby reducing its activity. This is currently the most advanced biologic therapy for the treatment of Stevens-Johnson syndrome and toxic epidermal necrolysis [15–17] ⑤Use the steroid rationally and reduce dosage slowly (Fig. 5); ⑥ The initiation of mycophenolate mofetil should involve a gradual dosage escalation, closely monitored in conjunction with the patient’s postoperative blood routine, peripheral blood lymphocyte count, and lymphocyte subpopulation analysis. Regular monitoring of these parameters is essential to assess the patient’s response and adjust the medication accordingly. ⑦ Tacrolimus: Before initiating tacrolimus therapy, it is essential to evaluate the patient’s renal function. Consider delaying the start of tacrolimus in cases of impaired renal function. Regular monitoring of renal function throughout tacrolimus therapy is essential to optimize drug management and minimize the risk of nephrotoxicity.

Toxic epidermal necrolysis dermatitis severely damages the protective barrier of the skin, increasing susceptibility to skin infections, as well as potential lung and bloodstream infections from skin-colonizing bacteria. This directly impacts the patient’s prognosis. In addition to reinforcing antimicrobial drug prophylaxis, it is essential to implement active skin care practices, adhere to strict aseptic protocols during medical procedures, and maintain an optimal level of immunity. Adopting an individualized immunotherapy plan, enhancing immune monitoring, and optimizing the use of postoperative immunosuppressive medications are essential to strike a balance between anti-rejection and anti-infection strategies Table 1.

**Table 1:** Patient’s Perioperative Laboratory results for heart transplant. ALT: Alanine aminotransferase; AST: Aspartate Aminotransferase; TBIL: total bilirubin; CREA: creatinine; BUN: blood Urea Nitrogen; BNP: B-type natriuretic peptide; tn T: troponin T

Laboratory indicators	Admission	Surgery	D1	D2	D3	D4	D7	D10	D23
Leukocyte, (10^9^/L)	9.75	13.78	14.08	13.3	15.54	16.33	8.48	9.63	5.4
Red blood cells, (10^12^/L)	5.18	2.25	2.71	3.24	3.41	3.05	3.43	3.39	2.28
Platelets, (10^9^/L)	81	75	54	41	30	77	56	70	40
Lymphocyte, (10^9^/L)	1.22	0.7	0.37	0.46	0.22	0.38	0.26	0.36	0.69
ALT(U/L)	13		26	25	18	14	10	9	10
AST(U/L)	20		133	132	39	22	13	14	17
TBIL, (umol/L)	11.2		13	14.8	13.6	16	14.7	13.2	16.7
CREA, (umol/L)	190.25	181.33	211.14	272.89	324.82	263.43	279.28	333.93	137.7
BUN, (mmol/L)	22.55	29.2	25.02	31.54	36.21	28.48	38.84	45.05	23.3
BNP, (pg/ml)	1427		1717	2386		1034	1485	1724	3435
TnT, (pg/ml)	58.5		2231	1740		923.5			288.9

## Data Availability

No datasets were generated or analysed during the current study.
